# Prediction of recurrent stroke and myocardial infarction after stroke: a systematic review of clinical prediction models

**DOI:** 10.1186/1745-6215-14-S1-O76

**Published:** 2013-11-29

**Authors:** Douglas Thompson, Gordon Murray, William Whiteley

**Affiliations:** 1 Edinburgh MRC Hub for Trials Methodology Research, University of Edinburgh, Edinburgh, UK; 2 Division of Clinical Neurosciences, University of Edinburgh, Bramwell Dott Building, Western General Hospital, Edinburgh, UK

## Background

Prediction models for recurrent ischaemic stroke or myocardial infarction (MI) after ischaemic stroke may be useful in targeting treatment. We aimed to systematically review the available prediction models. We studied (i) the methodological quality of the models and (ii) their related measures of performance.

## Methods

We searched Medline, EMBASE, reference lists and forward citations of relevant articles from 1980 to the 19th of April 2013. We included articles which developed a multivariate statistical model to predict recurrent stroke and MI after ischaemic stoke. We extracted data in duplicate using a validated data extraction form. We assessed model quality using pre-defined criteria and aimed to pool performance metrics (calibration and discrimination) using random-effects meta-analysis.

## Results

We identified twelve model development studies and eleven evaluation studies. Investigators often did not report effective sample size, regression coefficients, handling of missing data; typically categorised continuous predictors; and used data dependent methods to build models (e.g., univariate screening of predictors). Four models were evaluated. The pooled area under the receiver operating characteristic curve (AUROCC) estimate for the Essen Stroke Risk Score (ESRS) was 0.60 (95% CI 0.59 to 0.62, ten studies), for the Stroke Prognosis Instrument II (SPI-II) was 0.62 (95% CI 0.60 to 0.64, nine studies) and a single study of the Recurrence Risk Estimator at 90 days (RRE-90) was 0.72 (95% CI 0.56 to 0.88, one study) and of the Life Long After Cerebral ischemia (LiLAC) was 0.65 (95% CI 0.61 to 0.69, one study).

## Conclusions

The available models for recurrent stroke discriminate only modestly between patients with and without a recurrent stroke or MI. Performance may be improved by addressing commonly encountered methodological flaws.

